# A Survey on Trust Management for WBAN: Investigations and Future Directions

**DOI:** 10.3390/s20216041

**Published:** 2020-10-23

**Authors:** Samiha Ayed, Lamia Chaari, Amina Fares

**Affiliations:** 1 Institute Charles Delaunay-ERA, University of Technology of Troyes, 10300 Troyes, France; 2 Digital Research Center of Sfax (CRNS), Laboratory of Technology and Smart Systems (LT2S), University of Sfax, 3021 Sfax, Tunisia; lamiachaari1@gmail.com (L.C.); amounafs@gmail.com (A.F.)

**Keywords:** intra-WBAN, inter-WBAN, trust management

## Abstract

The rapid uptake of the Wireless Body Area Networks (WBAN) and their services poses unprecedented security requirements. WBAN are evolving to support these requirements. Fulfilling these tasks is challenging as their mobile context is increasingly complex, heterogeneous, and evolving. One potential solution to meet the WBAN security requirements is trust management that helps to reach a more secure and adaptable WBAN environment. Accordingly, this article aims to serve as a brief survey of trust management approaches within intra-WBAN and inter-WBAN. For that, we first summarize trust management concepts, discuss WBAN challenges and classify the attacks on WBAN trust management models. Subsequently, we detail and compare the existing trust based approaches in a WBAN context. We pinpoint their limitations and provide a new classification of these different approaches. We also propose a set of best practices that may help the reader to build a robust and an efficient trust management framework. We complete this survey by highlighting the open future directions and perspectives for research.

## 1. Introduction

The huge advances in new technologies and in the IT domain have directly served and positively impacted the e-health domain. The concrete application for that is the Wireless Body Area Networks (WBAN) adoption. The WBAN are largely deployed through different use cases. They are mainly used for remote diagnostics, also called tele-homecare, or for monitoring patients’ biological signals (such as temperature, blood pressure, heart rate, etc.) during long periods of time. They are also employed in combating diabetes, dementia, falls, congestive heart failures, asthma and infertility alongside several other medical contexts that require exhaustive attention and punctual responses. WBAN serve to better monitor patient’s health in real-time and react as much as possible in case of emergency [1]. For example, in the context of a heart attack, the body sensor network of the patient becomes busy by reading many medical measures at the same time. The collected data during a very short period of time should be transmitted for remote high intensive monitoring. Thus, WBAN require high accuracy and reliability, low cost and low power consumption, low End to End (E2E) delay due to their real-time aspects and high level of security, privacy and trust.

The WBAN can be also used for sports to control, for example, the vital signs of an athlete during their activity. Another interesting application of WBAN is their deployment in a crop’s environment to monitor crop health. They can be also deployed to mainly control the health of rare species to preserve them. In future, their deployment can be enlarged to the entertainment, lifestyle, environmental intelligence, ubiquitous computing, military or security fields [2,3,4,5].

Compared to the generic Wireless Sensors Networks (WSN), WBAN are characterized by the human involvement, mobility, small scale and a very high data rate. On the contrary, WSN are automatic and standalone, have a fixed or distributed deployment, have a large scale and their data rate depends on the applications reliability. Table 1 presents the different characteristics and requirements of both of them. Unlike WSN, the WBAN are composed of sensor nodes characterized by heterogeneous requirements in terms of delay and data rate [6]. Table 2 gives an idea about these diverse requirements of some commonly used sensors within the e-health domain.

In a medical context, the WBAN manage sensitive data related to the patient health. Thus, their requirements in terms of Quality of Service (QoS) and security are very high. Many research works [7,8,9,10] focused on the quality of services provisioning in order to satisfy WBAN application constraints. Besides, to ensure the security within WBAN, the security mechanisms to be deployed have to take into account the WBAN nodes characteristics. For example, concerning the delay-sensitive data, the security mechanisms, such as the trust management, should be lightweight to avoid affecting the WBAN applications QoS.

The WBAN deployment is technically possible through the use of intelligent devices, such as telehealth response watch, Remote Patient Monitoring (RPM) sensors and general examination camera, useful for physiological data sensing [11,12,13]. These devices are implemented on, in and around human body. They are connected and capable to communicate with interactive software for the analysis and presentation of the captured data through a local processing unit (LPU). The LPU serves as the gateway between the access points (APs) and the physiological sensors implemented on the human body. The WBAN sensors can also interact with the patient’s environment such as the medical or the non medical sensors existing in their location environment [14]. There are two communication schemes for WBAN. (1) Intra-WBAN: it only considers the communications within a cluster of body sensors. All these nodes can communicate with the WBAN’s central entity LPU. The LPU is considered as a cluster head of communicating nodes. (2) Inter-WBAN: for this scheme we consider the communications between different LPU entities that represent the central entities of different communicating WBAN. Based on these two communication schemes, the deployment of WBAN presents mainly four possible scenarios defined as follows:

- On-body: it concerns the communications between two different parts on the same human body surface.
- In-body: it concerns the communications from inside the human body to the body surface.
- Off-body: it concerns the communications from the human body surface to any equipment closely located around 3 m of the human body.
- Body-to-body: it represents the inter-WBAN communications defined between two subject’s bodies.

**Figure 1:**
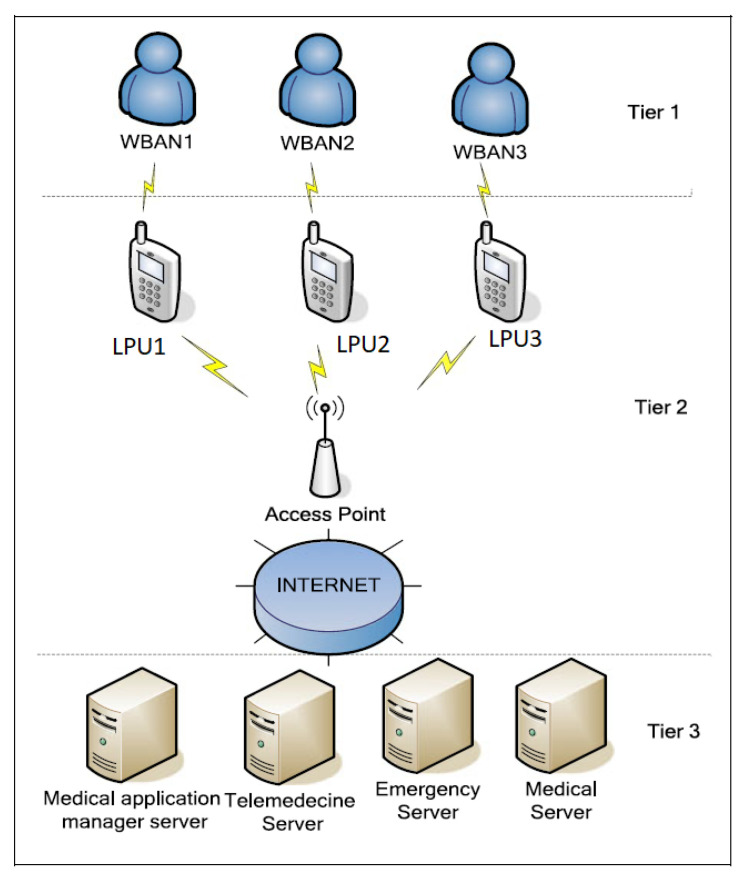
WBAN architecture example.

These scenarios can be defined based on a three or a four layers architecture including the intelligent medical devices and the gateways characterized by limited resources (energy, memory, computation, transmission power). A comparison of the technologies that can be used for such architecture can be found in [14]. In a WBAN architecture, the LPU pre-processes the collected data and transmits them (via the internet) to the cloud in the case of a 4-tiered architecture [15] or to a remote server in the case of 3-tiers architecture. Figure 1 gives an example of the 3-tiered architecture.

### 1.1. Problem Statement

WBAN manage sensitive data related to vital signs of patients. Furthermore, these networks present very demanding requirements in terms of energy consumption and security. The collection of data on different network nodes and the use of a wireless communication expose the WBAN to different possible attacks that may lead to critical situations related to the patient’s life. Thus, the security of the sensitive exchanged data as well as the security of their treatment are primordial challenges. Indeed, the unauthorized entities with malicious behavior try to use private data of the patients to carry out an attack. Thus, the non-authorized access should be forbidden and the communications should be secure. The main security properties that should be ensured are availability, confidentiality, authenticity, integrity and data freshness [16,17]. To achieve the security requirements, various approaches can be deployed [18,19]. Many surveys have dealt with the security approaches to secure WBAN ([20,21,22,23,24,25,26,27,28,29,30,31,32,33,34,35,36,37,38,39]). However, none of them has addressed the trust management for WBAN. Furthermore, numerous surveys exist about the trust management in WSN ([40,41,42,43,44,45,46,47,48,49,50,51,52,53,54,55,56,57,58,59,60]). The application domain of these surveys considers the Mobile Adhoc Networks (MANETS), IoT (Internet of Things) or WSN. Nonetheless, none of them has considered the WBAN as an application domain.

In this work, we consider the security issue from the trust point of view. Hence, we focus on trust management approaches in WBAN. We discuss the challenges and classify the attacks related to trust management in WBAN. Then, we deeply study and analyze the relevant and recent contributions and investigations spotlighting trust management mechanisms for intra and inter WBAN.

### 1.2. Main Contributions and Paper Organization

The trust management issue has attracted many researchers. A large state of the art and various surveys exist around this topic. Despite of these numerous surveys, none of them has dealt with the trust management in the context of WBAN. In this paper, we propose a survey about trust based approaches for WBAN. The contributions of this manuscript are:

- We analyze the WBAN challenges in terms of trust management.
- We present the different attacks that may occur in the context of WBAN as well as the trust management requirements related to these attacks. We categorize these attacks in three classes.
- We classify the existing approaches in two categories: the intra-WBAN and the inter-WBAN approaches and detail their specific contributions. Furthermore, we compare them and point up their different limitations.
- We propose a new classification of the existing approaches based on a set of criteria: trust properties, trust objectives, trust techniques and trust computation methods.
- We propose a set of best practices that may help the reader to define the relevant steps for building a robust and an efficient trust management framework.
- We pinpoint the open future directions for dealing about trust management in the context of WBAN.

**Figure 2:**
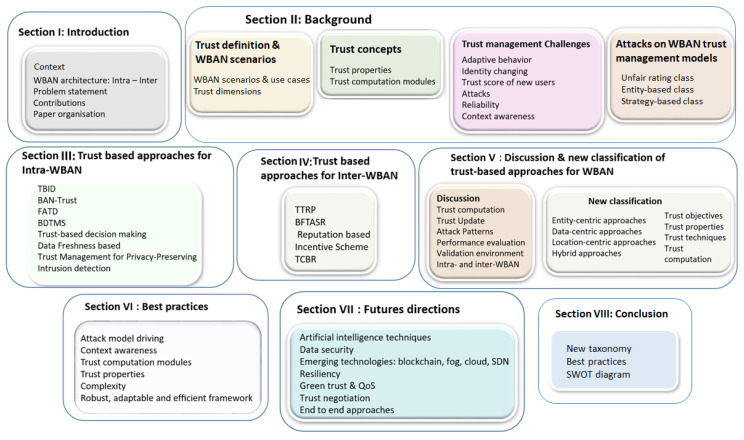
Survey overview.

The rest of the paper is organized as follows: Section 2 presents the trust management concepts and discusses WBAN trust management challenges as well as the WBAN related attacks. Section 3 details the approaches proposed in the context of intra-WBAN. Section 4 deals with inter-WBAN approaches. In Section 5, we compare and discuss these different approaches. Further, we propose a new classification of them and pinpoint their different limitations. Section 6 proposes a set of best practices to follow when building a trust management framework. In Section 7, we outline the main future directions that need more attention about the trust management in WBAN. Section 8 concludes the paper. Figure 2 presents the survey overview.

## 2. Background

### 2.1. Trust Management Concepts

To understand the trust management approaches proposed in literature to secure WBAN, we start by giving a clear definition of the trust concept.

First, we should differentiate between trust and trustworthiness. This latter consists on the state or quality of being trustworthy or reliable. Trust is sometimes confused with reputation. In networks, the reputation of a node is the idea or opinion that others have made about that node and how they consider it. This reputation can be built during different communications with that node and based on its behavior.

Generally, the trust definition can be related to the discipline for which it is applied. For example, in sociology, the trust concept is directly related to the persons and serves to build the social relationships. However, trust in economics domain is built on the hypothesis that humans are rational, and a strict utility enlarges their own interests or incentives. In the domain of networking security, the trust management has been introduced to enhance the security of networks through maintaining a good trust level within the network communications and nodes relationships.

A trust relationship is defined between a truster and a trustee. We define the trust as the confidence level that a truster associates to the trustee. It is a subjective probability that is varying from 0 (completely distrusted) and 1 (completely trusted). Thus, we consider that the use of the trust concept within WBAN may have three different dimensions:

1. Device based dimension: the trust is the confidence level that a device puts on another device of the network.
2. Data based dimension: the trust is the confidence that a network node associates to the received data in a WBAN.
3. Human based dimension: the trust is the confidence level that the patient’s WBAN can grant to the environment that surrounds it.

**Figure 3:**
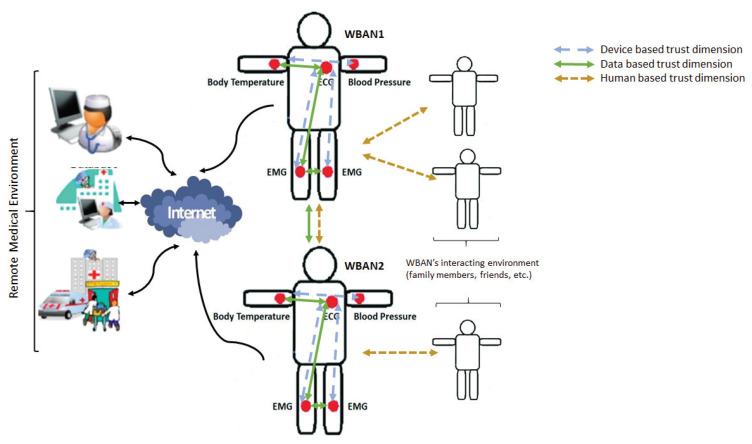
Trust dimensions.

Figure 3 gives an architecture of these different trust dimensions.

The scope of this survey includes approaches dealing with these three trust dimensions. The differences appear only in the sides of the trust relationship (truster, trustee).

The trust has many properties. The trust can be:

- Direct: the truster and the trustee have a direct communication and the trust value is calculated and deduced from this direct relationship.
- Indirect: the truster and the trustee do not have a direct link. The trust value about the trustee is calculated based on the recommendations propagated from different nodes of the network to the truster.
- Subjective: the trust is considered as subjective when it is calculated based on a personal opinion.
- Objective: the trust is considered as objective when it is calculated based on some specific parameters related, for example, to the device’s QoS.
- Local: the trust value between a truster and a trustee is only valid between these two nodes. The trustee may have another different trust value from another truster in the network.
- Global: each node has a unique trust value which is known by all the other network nodes.
- Asymmetric: it means that even when a node I trusts a node J, then the node J may not trust the node I.
- History-dependent: the trust is calculated based on the nodes’ behavior in the past.
- Context-dependent: the trust value between a truster and a trustee may vary from a context to another depending on specific events or conditions.
- Composite: the trust value may be composed of many parameters like honesty, reliability, security, etc.
- Dynamic: the trust value should be updated depending on the changes that may occur in the topology, the properties of the network or the conditions environment in which it has been calculated.

The trust management computation process has been described in many works ([43,44,45,50,60]). In this subsection, we recall the different trust modules that can be considered for a trust management process. A detailed classification of these modules can be found in [55].

- Trust composition: it includes the parameters that may be used to calculate the trust. In the context of the IoT, the two known parameters used for this purpose are the QoS and the social trust. The QoS represents the IoT device performance such as competence, cooperativeness, reliability and task completion capability. The social trust considers the relationship between different IoT owners. This parameter includes the intimacy, honesty, privacy, unselfishness, centrality, and the connectivity. It is especially considered in the case of social IoT systems.
- Trust propagation: this module defines how the calculated trust value about a specific node is communicated to other nodes of the network. Two approaches are possible. (1) Decentralized: the trust value is propagated to all nodes of the network, (2) centralized: we consider in this case that any node trust value is only sent to one central entity representing the network trusted entity. This entity is responsible for managing trust in the whole network based on a specific algorithm.
- Trust aggregation: when a node receives many trust values about another node, the trust aggregation module should be applied. The role of this module is to combine these different inputs in order to generate a unique trust value to be considered for that node. Many techniques may be used to deduce this new value such as fuzzy logic, Bayesian inference, etc.
- Trust update: during this module, the trust value is updated/recalculated. It is related to specific conditions that may occur on the network topology or some changes that happen on the nodes’ behavior. In this case, we deal about event-driven scheme. When the trust values should be updated periodically, we deal with a time-driven scheme.
- Trust prediction: it consists on using different techniques to predict the possible trust values of different network nodes. The prediction process may be related to some events like the history or the behavior of different network nodes.
- Trust formation: this module used the trust properties to calculate the trust. When only one trust property is considered, then we deal with single trust. However, when many properties are correlated to deduce the trust property, we deal with multi-trust.

**Figure 4:**
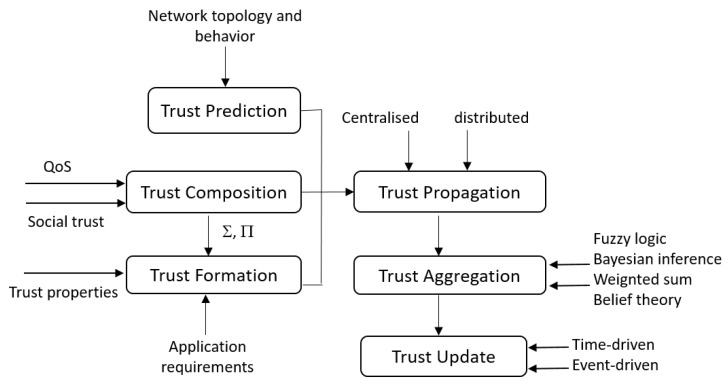
Trust computation modules.

To define and represent the relationships between the trust management modules and to show their interactions, we propose the Figure 4. Figure 4 correlates the different trust phases to define the trust cycle that should be considered when a trust management strategy is used to improve the security of an IoT network in general and a WBAN in particular. Figure 4 differs from the classification proposed in [55] by including the order of the different phases and shows the input/output relationship between them.

### 2.2. WBAN Trust Management Challenges

In the WBAN architecture, different network nodes gather and exchange data between them and with the LPU. These data are then sent to the medical staff or to the medical database. During the whole process, we have to be sure that all the collaborating entities are honest and none of them represent a failure point for the whole system. The trust management is one of the solutions that may be used to achieve this requirement within the WBAN architecture. Thus, all the nodes as well as the LPU should be reliable, secure and trustworthy. In this context, WBAN present a set of challenges that we outline in the following and have to be addressed in order to make the use of WBAN as secure as possible.

- Adaptive Behavior: the behavior control of the nodes placed on the body helps to detect the changes that may suddenly occur. The detection of the unfair behavior is based on the history of the node habit. The changing behavior of some nodes makes this detection phase difficult and so leads to unreliable information. Thus, the trust management mechanism should be capable to calculate the trustworthiness of the nodes past behaviors. The trustworthiness value should rely on the amount of interactions between the entities.
- Fair treatment of new users: when a new node joins the network where a trust management approach is applied, it should have an initial trust value to integrate the trust process. The assignment of the initial trust value for new devices is challenging. On the one hand, assigning a low trust value may affect the node performances notably for honest nodes. On the other hand, trusting a new device and assigning a high initial trust value may disturb the network behavior when the node turns out to be malicious. A process to evaluate the initial trust value of a node is required when a new device joins the network. That process may be based on some node’s credentials or on a specific authentication procedure.
- Context awareness: the trust computation should take into account the different context criteria that may be application dependent, network dependent or node dependent. These information make the trust calculation more accurate and make the trust value more realistic and relevant.
- Changing identity: One of the frequent challenges that a WBAN trust management framework should face is the varying of the node identity. Indeed, a node can initiate various identities and changes them frequently. When these identities are fake, we deal with the sybil attack [29]. Malicious nodes use this technique when they are assigned a low trust value. Authors in [29] proposed a Sybil Attack Prevention Algorithm for Body Area Networks (SAPA-BAN) algorithm to prevent against sybil attack in the context of WBAN.
- Reliability: the WBAN need to be reliable since they manage sensitive data and may deal with emergency situations related to the patient health. The reliability is ensured when the data amount involved in calculating trust is important. When the data sources are various, the error rate decreases. This challenge has been addressed in [30]. The authors proposed Reliable Ad-Hoc On Demand Distance Vector (RelAODV) to enhance the WBAN reliability.
- Trust score of new users: independently of the initial trust values of the new comers, the communications within the WBAN should prioritize the familiar nodes that are judged honest. That may enforce the reliability of the network and help the WBAN to be trustworthy as longer as possible. If they are trustworthy, these new nodes will rapidly achieve high trust levels and will be familiar nodes further.
- Large set of attacks against trust management: the WBAN are exposed to a very large spectrum of attacks. The trust management framework should prevent the maximum of these attacks. The most of these attacks are presented in the next subsection.

### 2.3. Different Attacks on WBAN Trust Management Models

To deal with the attacks related to the trust management models, we classify them in three categories: (1) unfair rating class based on changing the recommendation or the reputation values about a node or even providing erroneous values about the victim node, (2) entity-based class where the malicious node is based on changing its identity frequently to make its attack implicit and (3) strategy-based class where the attacker follows a specific behavior in order to make its attack undetectable.

#### 2.3.1. Unfair Rating Attacks

- Ballot-box Stuffing: it occurs when a node gives more than one positive opinion about the victim node. The multiple recommendations aim to reinforce the positive opinion about that node.
- Bad-mouthing attack: this attack consists on giving bad feed-backs on a node even when it is honest and behaving correctly. The aim of this attack is to decrease the reputation and so the trust level of the victim node. The impact of such attack is critical on the trust management framework since the nodes classification (honest vs. malicious) will be erroneous.
- Collusion attack: it is based on a coalition of a set of nodes in order to decrease the reputation of the victim node. It is a collaborative attack performed by sending fake recommendations about a specific node.

#### 2.3.2. Entity-Based Attacks

- Whitewashing attack: when a device has a bad trust rating, it may re-initiate with another identity.
- Sybil attack: when a malicious node in the WBAN creates a large amount of fake identities we talk about the sybil attack. These fake identities are also called pseudonyms. They are used to get larger influence over the erroneous information on other nodes. To defend against this attack, the trust management mechanisms can reinforce the use of authentication mechanisms in order to make the registration of fake identities difficult.
- Newcomer attack: when a node can easily register as a new user, we talk about newcomer attack. That node creates a new ID in order to erase its bad history. To prevent this type of attack, the trust management systems propose to assign low trust values to new comers nodes. Thus, they will not have a great influence in making decisions. To increase their trust level, they have to prove their honesty by well behaving during a period of time.

#### 2.3.3. Strategy-Based Attacks

- On–off attack: trust systems are vulnerable to on–off attacks for which malicious nodes can opportunistically behave badly or correctly. In this attack since the node behavior is good from time to another, the misbehavior will be considered as an error. Thus, malicious nodes can remain trusted while behaving badly.
- Traitor attack: it refers to malicious devices that initiate trustworthy interactions with different entities and then use their trust levels to behave wrongly during a specific time period until their trust levels are decreased. This attack is mainly feasible in systems using binary feedback or those where the reputation values are not updated frequently depending on the user’s behavior. Hence, the malicious nodes may exploit their reputation for a long period.
- Grudge attack: in this attack, the malicious node, usually unauthorized, gains access to the network through another trustworthy node already belonging to the network. The malicious node uses a specific strategy to convince the victim to add it to the network.

Table 3 presents WBAN trust management requirements for these different attacks.

## 3. Trust-Based Approaches for Intra-WBAN

In this section, we study and examine the state of the art focusing on intra-WBAN trust mechanisms. In our investigation, we have been concentrated on recent trust approaches dedicated to WBAN or IoT healthcare systems. In the following, we summarize the main ideas and present the contributions of these approaches. Moreover, we analyse the existing proposals through highlighting their advantages and limitations. We also consider in our analysis a set of comparison criteria related to the used trust objectives, the trust properties, the trust techniques as well as the trust computation modules.

- TBID: Authors in [61] proposed a Trust-Based Intrusion Detection (TBID) System based on the following aspects:
  1. To compute the direct trust ($T_{D}$) based on the interactive nature to acquire the direct trust between the two neighbors.
  2. To consider three factors: energy trust ($T_{e}$), data trust ($T_{d}$) and communication trust ($T_{c}$).
    (1) $$T_{D}=w_{c}T_{c}+w_{e}T_{e}+w_{d}T_{d}$$
    where $w_{c}$+$w_{e}$+$w_{d}$ = 1
    (a) The energy trust is used to check the node capacity for achieving the required task. It is based on the comparison of the residual energy to a threshold.
    (b) The data trust calculated to validate whether a neighboring node is capable to forward the data.
    (c) The $T_{c}$ of a body sensor node is computed according to the successful and unsuccessful communications among the body sensor nodes upon the provided time period.
  3. To identify malevolent nodes based on ($T_{D}$) value. Therefore, malevolent nodes are detached from the network.
  Even though the authors have proposed a distributed trust management approach, they have based their scheme only on the direct trust built with a simple formula. They did not consider the various trust properties and so omitted the indirect and the history trust. Moreover, the composition and formation phases of the trust management process were not considered. However, the approach describes how the trust level is periodically updated. Besides, the approach evaluation is mainly based on the pattern of the bad mouthing attack.
- Body Area Network trust (BAN-Trust): Authors in [62,63] conceived an attack-resilient malicious node detection scheme for WBAN named “BAN-Trust”. The WBAN node behavior will first be observed and analyzed in the data analysis process. Then WBAN nodes are classified into potential malicious nodes (examined by malicious node detection) or into evidence nodes, which can then be used by a trust management module. Second, each node computes a recommendation trust using collaborative filtering according to the following process:
  1. For a WBAN with a set of q devices, each node $N_{j}$ computes a vector for the recommendation trust ratings that device $N_{j}$ makes for each $N_{i}$ in WBAN denoted as $VN_{j}$ = $[vn_{j1},vn_{j2},$ …$,vn_{jq}]$. Therefore, the trust ratings of each WBAN is a *q*×*q* matrix R where each element in the matrix, denoted $rN_{j}$, $N_{i}$, corresponds to the trust rating for node $N_{j}$ and another node $N_{i}$.
  2. The credibility of recommendations of node $N_{k}$ can be computed by the similarity (using the Cosine-based similarity metric) of the trust rating information between node $N_{j}$ and Node $N_{k}$.
  3. The value of the unknown trust rating $rN_{j},\;N_{i}$ is computed as an aggregate of the ratings of K most similar users for the same node $N_{i}$.
  4. Trusted neighbor selection: the top K most similar nodes are selected based on similarities between nodes. Then, the functional trust of each selected node is checked and only nodes that can fulfill their tasks as expected are accepted.
  5. Predicted trust calculation: the predicted trust rating of $N_{i}$ on $N_{k}$, denoted $T_{ik}$, is calculated based on Resnick’s standard prediction formula [64].
  Here, as in [61], the authors considered only the indirect trust. They used the collaborative filtering to focus only on the aggregation module and the update module. Moreover, similarly to [61], they considered the same attack pattern of the bad mouthing attack to evaluate their approach as well as a time-driven update of the trust process. However, the propagation, the formation and the composition modules have been omitted.
- Trust-Based DoS Mitigation Technique for Medical Implants in WBAN: Authors in [65] proposed a trust-based rate limitation to mitigate Denial of Service (DoS) attack in the Medical Implant Communication Service (MICS) network. Accordingly, the authors considered that the home is the most trusted environment, thus it has the higher threshold. The work environment is the next level of trusted environment where the maximum threshold value is reduced. A public place is the least trusted environment. At this level, the maximum threshold is further reduced. The drawback of this approach is the fact that the trust level is fixed and it is related only to the location without considering data or nodes. Contrary to all existing approaches, [65] is based on the human dimension of the trust concept. Indeed, it is directly related to the relationship that the person may have with their environment while calculating the trust level based on their location. Here also, authors considered only the direct trust. They evaluated their approach based on the DoS attack pattern using the calculation of simple formula. In addition, contrary to [61,62], they omitted the trust update process and only focused on the aggregation module.
- A trust based distributed intrusion detection mechanism [66]: Authors described the trust management aspects used to allow Destination Oriented Directed Acyclic Graph (DODAG) nodes building up trust relations with their neighbors involved in the routing messages through the network. Accordingly, four phases are suggested: (1) trust evaluation, (2) computation of the direct trust of neighboring member nodes, (3) trust value combination and (4) reputation management:
  1. Trust values are computed from positive (p) and negative (n) experiences with a trustee by the following metrics:
    (2) $$b=\frac{p}{\left(p+n+k\right)}$$
    (3) $$d=\frac{n}{\left(p+n+k\right)}$$
    (4) $$u=\frac{k}{\left(p+n+k\right)}$$
    The constant *k* is equal to one or two. In order to enable the nodes to rate their neighbors, a node can then listen to its neighbor’s transmission and rate them positively if they behave according to the Routing Protocol for Low-Power and Lossy Networks (RPL) protocol and negatively if they differ from it.
  2. This phase corresponds to the direct trust computation of a node *x* in another node *y* via monitoring messages sent by *y*. Therefore, a node *x* performs three checks: forwarding check, ranking check and version number check.
  3. The nodes forward their trust values to a cluster-head or to the border router that aggregates them to reputation values. The Subjective Logic defines a consensus operator ⊕ for Trust values aggregation. A bad reputation value indicates a node as a potential intruder.
  4. To manage reputation, the authors evaluated three approaches: Neighbor Based Trust Dissemination (NBTD), Clustered Neighbor Based Trust Dissemination (CNTD) and Tree Based Trust Dissemination (TTD).
  Similarly to [65], the authors considered only the aggregation process within their scheme using the subjective logic. However, contrary to the previous approaches [61,62,63,64,65], the proposed approach included the direct, the indirect and the historical trust. Moreover, the trust approach has a different objective directly related to the routing within the WBAN.
- A Lightweight Encryption Scheme Combined with Trust Management for Privacy-Preserving in Body Sensor Networks [67]: in this paper the authors considered the multi-level trust to propose different privacy-preserving strategies to users coming from a different trust set during transmission. They defined three different classes of users: (1) the family members of the patient, (2) the social network of the patient and (3) the strangers. Then, they attribute a level of trust, from high to low, to each of them. The authors defined three strategies to leverage the Personal Health Information (PHI) privacy disclosure and the high reliability of PHI process and transmission in m-Healthcare emergency: (1) lightweight and anonymous authentication, (2) ABE-based access control and (3) lightweight encryption. The chosen strategy depends on the information that should be encrypted: the patient identity, the whole PHI or only a part of it. They also introduced an efficient lightweight encryption for those users whose trust level is low. The simulation results show that the use of trust management makes the proposed scheme performances better than those presented in [68].
  The centralized approach presented here considers, similarly to [65], the human dimension of the trust concept through relating the trust concept to the persons interactions within the WBAN. Compared to [65] that presented a location-based approach, the authors in [67] included the privacy preservation of the exchanged data. Thus, the proposed approach is hybrid taking into account the data and the nodes composing the WBAN. It is updated when the environment of the WBAN changes. Likewise in [61,65], the authors considered only the direct trust.
- Fault Aware Trust Determination Algorithm for Wireless Body Sensor Network (WBSN) [69]: the authors propose to relate their trust computation process to the energy capacity of different nodes, to their mobility and to their reliability depending on the link strength between two nodes. Indeed, they consider the parameters about the battery terminal voltage, the receiver signal strength and the nodes speed of movement. Using these attributes, they define the energy trust, the reliability trust and the mobility trust using the following formulas:
  (5) $$E_{t}=a_{1}\sin\left(b_{1}x+c_{1}\right)+a_{2}\sin\left(b_{2}x+c_{2}\right)$$
  where $E_{t}$ is the energy trust and $a_{1},b_{1},c_{1},a_{2},b_{2},c_{2}$ are curve constants.
  The reliability trust:
  (6) $$R_{t}=e^{-\lambda d}$$
  where $R_{t}$ is the reliability trust, λ is the slope of the curve and *d* the distance between the sender and the receiver.
  The mobility trust:
  (7) $$Mt=\alpha \beta^{-\alpha }x^{\alpha -1}e^{-{\left(\frac{x}{\beta }\right)}^{\alpha }}$$
  where α and β are the shape parameter and scale parameters of the curve.
  Based on that, they propose a Fault Aware Trust Determination (FATD) algorithm. They define an overall trust and different trust levels. The nodes having high trust energy, less mobility and good reliability will be assigned a high trust level. Then, to deliver data, they calculate the most trustworthy route that includes better trustworthy nodes. Hence, they increase the network lifetime. To evaluate the proposed solution, they compare the performances of their proposal to the two existing protocols Low Energy Adaptive Clustering Hierarchy (LEACH) [70] and QoS-Aware Peering Routing for Reliability (QPRR). Their simulation results show a better performance concerning the network throughput and the network lifetime. Ref. [69] presented a decentralized approach that is mainly based on the trust composition module and updated when the nodes parameters change. All other trust phases as well as the trust properties were not exploited (aggregation, formation, propagation). Besides, the test environment was not detailed to better understand the parameters included in the evaluation process of the approach.
- A Novel Trust Evaluation Model Based on Data Freshness in WBAN [71]: To define their scheme, authors are based on the data freshness property of transferred information. Indeed, they consider different permitted delays associated to each type of forwarded data. For example, ECG data is considered critical for a heart patient contrary to the body temperature of an athlete. A threshold value is defined as the midpoint between the permissible delay of each type of information and the delay average of that information for all nodes of the network. The delay of specific node for forwarding data is compared to that threshold to decide if the node is malicious or nor. To evaluate the proposal, two scenarios about critical and non-critical data were considered.
  Contrary to all existing approaches, the approach presented in [71] is the only approach based on the data. The trust level does not concern the WBAN nodes but it is related to the exchanges data within the WBAN. For that, the authors considered only the trust update module and aimed to isolate the nodes managing non-fresh data from the network.
- Trust-based decision making for health IoT systems [72]: Authors are based on three parameters to define their trust based protocol: (1) The member’s healthcare classification, denoted (Z), that classifies members based on the patient health. If the patient has critical diseases, their level of health or index is low and from that we deduce their high vulnerability index, denoted Z ∈ [0, 1], that has to be superior than 0.8. (2) The reliability trust of the source (p): this parameter assesses the trustworthiness of different members, and (3) the probability of health loss, denoted *G*, measures the probability that the patient presents serious repercussions if some of their vital signs are altered. This probability is generated based on the *p* and *Z* parameters. The trust decision formula is defined on these three parameters as:
  (8) $$Z=p^{\gamma }\times {\left(1-G\right)}^{\omega }$$
  the parameters γ and ω are application-specific and fixed respectively to 2 and 1 in the paper. After formalizing their protocol design, the authors presented the evaluation results that show a high correct decision ratio even while the malicious nodes are increasing within the network. They also compared their proposed protocol to two scenarios to show its better performances. The first scenario does not consider the trust evaluation. The second scenario considers the trust calculation but without considering the member health parameter.
  Similarly to [69], the centralized approach proposed in [72] is based on the trust composition module. Besides, it integrates a human dimension of trust like in the case of [65,67]. However no trust property has been considered.
- BDTMS (Binomial Distribution-based Trust Management Scheme for Healthcare-oriented Wireless Sensor Network) [73]: To calculate the trust value of a node, the authors are based on indirect information collected from common neighbors of that specific node. Then they apply aggregation formula on that information to obtain the trust value. They use a binomial distribution to simulate the interaction between nodes. From that, they deduce the trust value of node *i* to node *j* as follows:
  (9) $$T_{i,j}=\frac{a}{\left(a+b\right)}$$
  where a+b is the interaction number between the two nodes i and j, a is the number of cooperation and b the number of non-cooperation.
  The aim of the solution is to encounter the On-Off attack. In such attack, a malicious node behaves alternately as normal and abnormal node. To detect this behavior, the authors consider time intervals to supervise the variation of the node trust value. Depending on the variation of that value, an interpretation about the node behavior is constructed. Based on the simulation results, BDTMS outperformances the Beta Reputation System for Sensor Networks (BRSN) [74] and the Time-Window-Based Resilient Trust Management Scheme (TRTMS) [75] schemes, considering a higher detection accuracy and a less detection time.
  The presented approach in [73] is the only approach that treated the On-Off attack pattern, considered as an insider attack. For that, it exploited the propagation, the aggregation and the update modules using only the indirect trust.
- A distributed trust evaluation model and its application scenarios for medical sensor networks [76]: In order to detect and exclude malicious nodes, the authors propose to calculate trust taking into account the transmission rate and leaving time parameters. They consider three trust values: (1) the direct trust calculated for the direct neighbors, (2) the recommended trust calculated for indirect neighbors and (3) the historical trust calculated based on the nodes’ behavior for the previous period of time. The overall trust value of a node A on a node B for a specific action act is then deduced based on the following formula:
  (10) $$T\left(A:B,act\right)=\alpha \times T^{y}\left(A:B,act\right)+\gamma \times T^{h}\left(A:B,act\right)$$
  where $T^{y}\left(A:B,act\right)$ is the direct (respectively, recommended) trust value and $T^{h}\left(A:B,act\right)$ is the historical trust value for direct neighboring nodes (respectively, indirect neighboring node) during the previous unit of time. The aggregation of the overall trust value for all performed actions is given by the following formula:
  (11) $$T_{AB}^{total}=\epsilon_{i}\times T\left(A:B,act_{1}\right)+\ldots +\epsilon_{p}\times T\left(A:B,act_{p}\right)$$
  where $\epsilon_{i}$ are application dependent coefficients. To evaluate the performances of the proposed approach, the authors measure the average packet reception ratio that gives good results.
  Like the proposal of [65], the proposed approach in [76] considered the DoS attack pattern. For that, it used the trust aggregation module based on the direct, the indirect and the historical trust. This centralized approach is based on a time-driven update of the trust level associated to nodes. Thus, the trust management process is periodically updated to integrate the environment changes.

## 4. Trust-Based Approaches for Inter-WBAN

In this section, we focus on the trust management approaches in inter-WBAN systems. The existing approaches for the inter-WBAN present a common point about the trust management objective. Indeed, the existing approaches, that we detail their main ideas in the following, concern the routing algorithm. The trust concept serves in this case to determine the most secure routes that should be selected to forward data. Thus, the considered trust level is mainly associated to nodes that will participate in transmitting packets. We conclude in this context that the trust concept is used at the routing level compared to the intra-WBAN approaches.

- Trust and thermal aware routing protocol (TTRP) for wireless body area networks [77]: TTRP is a three-phases routing protocol based on introducing trust to define the routes: (1) trust estimation, (2) route discovery and (3) maintenance phase. The first phase of the protocol uses the direct and the indirect trust to evaluate the trustworthiness of intermediate nodes. Then, comes the route discovery phase based on the trust estimation initially calculated to select the nodes involved in defining the routes. These nodes should not be a hotspot when they are involved in a route. The best path is calculated through a composite function defined as:
  (12) $$CF=\omega_{1}*trust+\omega_{2}*temp$$
  where $\omega_{1}$ and $\omega_{2}$ are respectively the proportion weights of trust and temperature.
  The best route is the one presenting the smallest value of the composite function. If one of route nodes become a hotspot during inactive route communication then, the maintenance phase is responsible of re-initiating the route discovery phase. The protocol aims to have only trusted routes by restricting hotspot/misbehaving nodes to be part of that trusted routes.
- BFTASR (Biometric Fusion based Trusted Anonymous Secured Routing protocol) [78]: Authors proposed a trust based routing protocol called BFTASR. The protocol considers that all nodes participate to calculate an objective trust value about all other nodes. Based on that value, the node can choose how to perform cryptographic operations during the routing process. This process is based on an onion routing defining three types of nodes: the entry nodes, the exit nodes and the other nodes. Entry and exit nodes have the highest trust level. Based on this node classification, a multi-level encryption is applied based on the node type. The use of biometric characteristics in anonymous authentication of trust based secure routing has many advantages. It not only reduces computational complexity but also improves the power efficiency. The performance evaluation of BFTASR shows that the trust based approach for the routing protocol improves the throughput, the ability to identify packet dropping attack, the packet loss ratio and the end-to-end delay, compared to Ad-Hoc On Demand Distance Vector (AODV), Anonymous On-Demand Routing (ANODR) and Anonymous Secure Routing (AASR).
- Reputation based Incentive Scheme for Secured Data Privacy in Wireless Body Area Network Communication [79]: authors proposed a new incentive based scheme to classify the trustworthy nodes. Each node of the network has a reputation calculated based on the following formula.
  (13) $$R_{SNi}=\frac{DP_{T}-DP_{D}}{DP_{N}}$$
  where $DP_{N}$ is the total number of received data packets, $DP_{T}$ is the number of successfully transmitted data packets and $DP_{D}$ is the number of dropped data packets.
  Users with higher incentive value are selected as nodes responsible for the data packets transmission. This classification is the result of the repute derivation based Incentive Algorithm that checks if the reputation value is increasing or decreasing and then deduces if the node may be malicious or not. Then, an aggregation process takes place based on the selection of data packets with minimum tolerance delay based on the following formula:
  (14) $$min\left(\tau_{i}\right)=min\left(\sum_{k=0}^{k=h}\tau_{k}\right)$$
  where $\tau_{k}$ is the delay of the hop *k* and *h* is the number of hops.
  The aggregated data is considered as the input of a compressed sensing process in order to perform encryption and decryption using a sensing matrix for providing privacy. This latter phase aims to improve the privacy rate of data transmission. The performance evaluation results show that the proposed RDI-SSDA (Repute Derivative Incentive and Sparse Sampled Data Aggregation) scheme lead to a higher throughput by 34% compared to the attribute-based encryption and signature scheme [80] and by 15% compared to PPM-HDA (Privacy-preserving and multifunctional health data aggregation) [81]. Moreover, RDI-SSDA, compared to the same schemes improves the data aggregation efficiency by 27% and 15% respectively. Concerning the data privacy level, it is improved compared to the same schemes by 17% and 8% respectively.
- An Optimal Trust Aware Cluster Based Routing Protocol (TCBR) [82]: in this work authors define their proposal in three steps: (1) proposing a clustering approach based on improved evolutionary particle swarm optimization (IEPSO) that defines how to select the cluster head, (2) proposing a fuzzy based trust inference model to choose the most trustworthy route to deliver messages, and (3) defining a self-adaptive greedy buffer allocation and scheduling algorithm (SGBAS) to decrease the quantity of traffic among network nodes. The nodes trust value is calculated by combining three parameters: the residual energy, the link expiration time and the received signal strength. The trust formula for a sensor node $s_{i}$ is defined as follows:
  (15) $$Output=\frac{\sum_{i=1}^{l}b_{i}\times s_{i}}{\sum_{i=1}^{l}s_{i}}$$
  where $b_{i}$ is the terminating quality of a fuzzy variable. Then, based on the formula results, different trust levels are defined and so used to define the most trustworthy path that contains nodes with the highest trust values. To evaluate their proposal, authors consider the nodes energy consumption, the latency, the delivery ratio, the network life span, and the model scalability. The better TCBR performances have been presented through a comparison with other clustering based routing schemes.

The inter-WBAN approaches [77,78,82] are associating the trust concept to the WBAN nodes. The approach presented in [79] is the only one that includes the transmitted data within the trust calculation process. Since the main objective of the trust management in inter-WBAN is to secure the routing, the forwarded data should be a mandatory dimension to be considered. This dimension may include the integrity property, the confidentiality as well as the privacy.

## 5. Discussion and New Classification of Trust-Based Approaches for WBAN

### 5.1. Discussion

**Figure 5:**
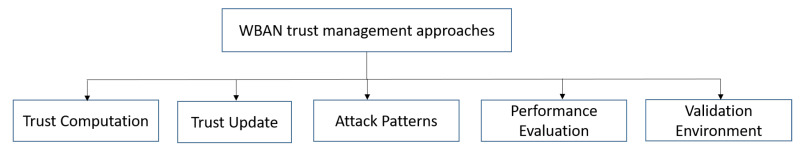
Comparison criteria for WBAN trust management approaches.

To summarize the different approaches discussed in the previous section we define five criteria that we schematize in Figure 5.

Based on those criteria, Table 4 and Table 5 recap the WBAN trust management approaches. The first analyze that we make for the existing works let us notice that there are only few works that have considered the inter-WBAN architecture. Most of the existing approaches deal with the intra-WBAN communications. However, the inter-WBAN communications may lead to new attacks through increasing the attack surface and introduce new challenges like the end to end delay. Furthermore, most of the considered approaches do not explain how the initial trust value is fixed or calculated. Hence, the proposed approach results may differ when we consider an optimistic scenario for which we initially admit that all network nodes are trustworthy and when we suppose from the beginning that all network nodes may be malicious. Moreover, we notice that the trust management has been used not only to prevent, detect and exclude malevolent nodes or to overcome some attacks but also to enhance the WBAN QoS. To go further in our discussion and analysis, we propose a new classification of the existing approaches that we present in the next section.

### 5.2. New Classification

**Figure 6:**
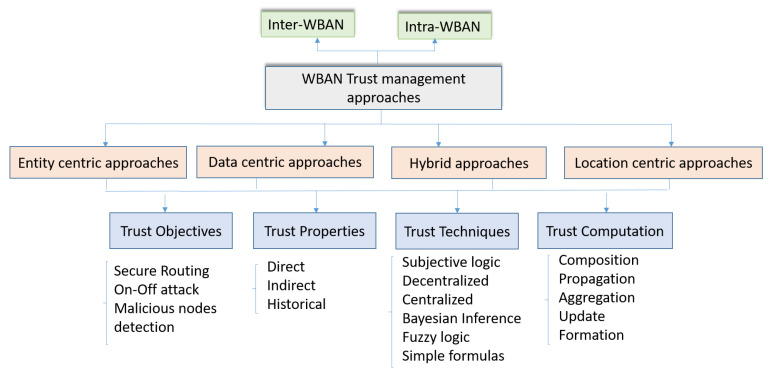
New classification for trust management approaches in WBAN.

To our knowledge, there is no survey that details the trust management approaches related to the specific context of WBAN. In this survey, we propose a new classification of the existing approaches in a WBAN context. In [83], the authors surveyed the different aspects related to the e-health environment: IoT-based technologies and their industry trends, the different network architectures and the platforms used in the context of e-health applications. It also presented the security and privacy requirements and challenges. However, they did not consider the trust management approaches. In [84], authors presented a taxonomy of trust and reputation schemes in healthcare systems that considers the attacks, the application area, the requirements and the architecture. WBAN are considered as an application area as well as pervasive social networking, ubiquitous health, telecare services, pervasive health, e-health systems and services availability in cloud. Hence, our survey differs from these surveys by focusing on trust management approaches specifically in the context of the WBAN. For that, as far as we know, this is the first survey covering this scope. To classify the existing trust management approaches, we propose the taxonomy presented in Figure 6. We classify the different approaches in four classes:

1. Entity-centric approaches: they focus on the legitimacy of the WBAN nodes. The focus for this class is made on the trustworthy level of nodes participating in delivering and routing data. For that the different types of the attacks considered in this category are related to the misbehavior of the WBAN nodes. The main objective through these approaches is to avoid, to detect and to exclude the malevolent nodes. Hence, they will not be able to affect the network performance and integrity or at least minimize their damage.
2. Data-centric approaches: for these approaches, the focus is on data. Indeed, we look to satisfy the data authenticity. We need to be sure that the provided data are accurate and correct. Some malicious nodes may affect the produced data in order to attack the network. This is more challenging when critical information, like ECG, are considered or when the remote control serves a critical patient (heart patient for example). Furthermore, the data privacy is also a challenging issue for this class. The main objective of trust management for this category is to ensure the integrity, the confidentiality and the privacy of the exchanged data.
3. Hybrid approaches: Combined trust management approaches are based on the trustworthiness of both entity and exchanged data, for better efficient trust computation. Entity trustworthiness assists data trust value assessment; The data content that has been evaluated to be reliable by many trusted entities is suggested as trustworthy to other nodes.
4. Location centric approaches: for this category the trust calculation process is based mainly on the nodes location. Indeed, instead to be based on the WBAN nodes attributes or to the exchanged data, the trust attributes are directly related to the environment in which the patient is present. For instance, if the patient is in a public environment, the trust level is considered low. When the patient is with their member’s family, the trust level is high.

For these four classes and to compare the reviewed approaches, we consider the following criteria: the trust objectives, the trust properties, the trust techniques and the trust computation. Table 6 provides the comparison that we performed based on our taxonomy (Figure 6).

### 5.3. Limitations of Existing Approaches

Based on the new classification that we propose in the previous sub-section, we deduce a set of limitations of the reviewed approaches that we summarize as follows:

- As we can obviously notice, the most of existing approaches are entity-centric. Despite the importance of the data either for the patient health or for the network performances, there are very few works that dealt with the data to apply trust management. This is more challenging when the produced data is used to remotely take decision or act on the patient.
- We note that the most of trust properties have not been considered during the trust calculation process. In fact, only some of the trust properties have been introduced to measure trust values for the existing approaches. For instance, no approach has considered properties like asymmetry, composition, dynamicity, context and history dependency.
- The trust computation modules have not been widely deployed. They were essentially based on the aggregation, the propagation and the update modules. In particular, the composition and the formation phases were under estimated. In the same way, the prediction phase was totally omitted, in spite of the advantages that it may bring to enhance trust management schemes.
- Concerning the trust techniques, the majority of works made use of simple formulas, some others have based their approaches on more usual techniques such as subjective logic, Bayesian inference, fuzzy logic and collaborative filtering. The artificial intelligence techniques have not been exploited to optimize the complexity of trust management process.
- For the inter-WBAN approaches, we notice that clustering techniques are under estimated and are only limited to be used in the context of routing protocols. However, many approaches exist in the context of trust management in the IoT proposing mechanisms for group formation based on the reputation values (reputation capital) of different devices [85]. These approaches can be facilitated by integrating the blockchain technology to certify the reputation capital [86]. Those approaches can easily be adapted to the context of WBAN for introducing the reputation in the trust calculation process for the inter-WBAN approaches.
- In most of the existing works, the complexity of the proposed approach is not evaluated and the energy consumption is not considered.

## 6. Best Practices

Our discussion about existing trust management approaches has focused on the trust management properties, objectives, computation and techniques. In this section we pinpoint the key elements that may help the reader to build and develop a reliable trust management framework for WBAN. These best practices take into account the limits of existing approaches and give practical recommendations about different steps of the trust management process.

1. Trust models for WBAN have to be independent of medical sensors constraints related to the memory usage, the computation performance and the transmission delay. The overhead that may be added related to the trust management process should not impact the communications quality.
2. The trust management framework should be an attack model-driving. It has to be directly correlated to the attacks detection. Moreover, when a detection is successfully performed, the calculation process has to be updated based on that event. Thus, the trust management process is more efficient against new attacks and progressively build its strategy to react against attacks that have been already carried on the network. Hence, we evolve towards an intelligent and autonomous trust management framework.
3. The trust models should be context aware. They should be dynamic, event driven and continuously updated. Furthermore, they have to consider the reputation calculation to get a more reliable trust value. The network evolution and the nodes’ behavior changes within time have to be supervised and directly correlated to the reputation and trust calculation processes.
4. The trust framework should consider the different trust computation modules to ensure an accurate evaluation of the trust value. During this process, we have to consider the application requirements, the different trust properties, the network topology and behavior. Considering only direct and indirect trust is insufficient. The trust calculation process has to be history dependent including positive and negative past feedbacks. Contrary to the existing approaches, building the trust process should make use of the different trust modules and more precisely the update, the prediction, the composition and the formation phases, actually under estimated. The most of proposed approaches are only considering the aggregation and the propagation modules.
5. The complexity of the calculation process should be low in order to not impact the QoS of the network, especially in the context of medical applications where the exchanged data is very critical and sensitive. The real time characteristic of such applications has to be preserved. Furthermore, the trust energy consumption evaluation should be considered to ensure the trust framework adaptation to the medical sensors capacities and constraints.

**Figure 7:**
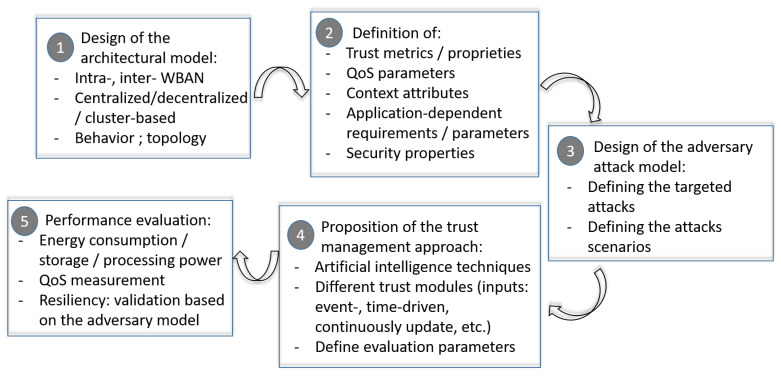
How to design and implement a WBAN trust management framework.

To pinpoint the key elements of building a trust management framework, we propose in Figure 7 the main steps required to get a reliable, adaptable and efficient framework.

## 7. Future Directions and Perspectives

1. AI techniques. As shown in our comparative study (Section 5), the existing approaches do not take real advantages from artificial intelligence techniques that are simply deployed. The advances made in that domain can be very useful to enhance the performance of trust management schemes. In fact, to calculate the trust value, more theories like Dempster–Shafer could be used. Moreover, techniques like game theory, neural networks and Q-learning can reinforce the trust management framework to detect malicious nodes based on the three detection approaches: the misuse-based detection, the anomaly-based detection, and the hybrid detection. Furthermore, more attention is required to deal with prediction module for the trust management process in WBAN. The proposals concerning this phase could be in correlation with collaborative filtering techniques that propose efficient algorithms to improve the prediction of nodes’ behavior. In sum, artificial-enabling techniques can be propitious for intuitive trust scores generation, approximation reasoning and better decision making. From another point of view, machine learning algorithms, for example Decision Tree, SVM, K-Nearest Neighbor and Random Forest, provide the data classification allowing the prediction of the value of a categorical variable (for example, the trust scores). This classification is possible through the construction of a model based either on numerical or on categorical variables. Moreover, these algorithms propose to learn and enhance the predictions from previous experiences. Thus, the learning phase can be widely used to perform a learning of the most know attack patterns to be able to easily detect at least the attempts based on that patterns. The machine learning algorithms have to be more explored to enforce the security in WBAN to detect effective anomalies. Furthermore, they need more attention in the context of trust management for WBAN since they can have various advantages: (i) the use of multi-dimensional attributes to calculate trust scores serves to have exact and reliable trust scores and leads to lightweight solutions; (ii) the use of multi-dimensional attributes makes the trust schemes easily scalable and model-independent if the WBAN need to be deployed in a dense environment (for example, within a huge hospital); (iii) the powerful machine learning tools offer an automatic learning and evaluation of the security risks.
2. Data Security. As we already explained, the trust management approaches can be classified in four categories: (i) entity-based approaches, (ii) data-based approaches, (iii) hybrid approaches and (iv) location-based approaches. The focus for future works has to be made on the data-based and hybrid approaches since most of the existing proposals in the context of WBAN deal with the entity-based approaches. Moreover, the managed data for WBAN are sensitive and considered as personal. Thus, the question about how to preserve the user’s or patient’s privacy should be tackled. Hence, the trust management framework has to make use of techniques that propose to protect the exchanged data. The two main pertinent examples that represent active research topics about that are the anonymization technique and the homomorphic encryption technique. The data management is more challenging especially within the context of the new regulations; e.g., General Data Protection Regulation (GDPR) that imposes specific compliance to respect the privacy and data security. In the same context, the conformity with the existing e-health standards should be ensured. Indeed, to ensure privacy, there are regulations, like the American Health Insurance Portability and Accountability Act (HIPAA) [87] and the Health Information Technology for Economic and Clinical Health Act (HITECH Act) [88], that detail precautions and rules to be respected to safely use, collect, store, share and manage sensitive patient information for administrative or communication needs.
3. Emerging technologies. The existing approaches for managing trust in WBAN are mainly based on some simple techniques of artificial intelligence to calculate trust. However, in recent years, many new technologies have been emerging and so challenging many scientific approaches. Software Defined Network (SDN), Blockchain, Fog, Edge and Cloud computing represent the foremost trends that awfully bring advantages to many domains and more specifically to the IoT domain. In the same way, these technologies can be auspicious to build more efficient trust management frameworks in the context of WBAN. Indeed, they can be used to support more dynamicity, flexibility, security and typically efficient resource utilization that enhances QoS. On the one hand, the adoption of the SDN [89,90], the edge and the fog computing [91,92] helps in optimizing resource allocation, providing lower delays and simple time complexity and hence reducing transmission costs. On the other hand, the cloud can facilitate the data management and processing for the trust management approaches in WBAN. When the WBAN are deployed in a dense environment, like a huge hospital, the cloud can serve as a solution to be adopted within the architecture that should be considered. Some works have made use of the cloud to ensure some specific security properties [93,94]. However, managing big data within WBAN is still in its infancy.
4. Blockchain technology: the blockchain technology has an opportune interest to be used for trust management in WBAN environment, by dint of its features [95,96,97,98]. Indeed, it is widely acknowledged that this technology can deal with centralization, security and privacy issues when storing, tracking, managing, and exchanging data. Hence, it may be widely used in data-centric and hybrid approaches cited above (see point 2). Even though the blockchain has not been exploited to manage trust within WBAN, it has been widely used to deploy other security mechanisms and ensure other security properties within the WBAN environment. For instance, ref. [99,100] adopt blockchain technology to protect patients privacy through a secure storage. Blockchain has also been used to enhance the security of routing algorithms in WBAN likewise the approach proposed in [101]. The deployment of the blockchain technology in trust management approaches for WBAN needs a big attention since it can be very useful specifically for the data-based approaches. Indeed, it allows the integrity verification of the data used in the trust calculation process, mainly for the artificial intelligence based approaches, through providing tamper proof data. Besides, it protects the privacy and the availability of these data during sharing and storage. Collecting the trust data, calculating trust scores and deciding about the trustworthiness level within the WBAN can be delegated to a blockchain based architecture. That can be a relevant proposal for the WBAN devices specifically with their limited calculation and storage resources.
5. Resiliency. Some of the reviewed approaches proposed an attack pattern to evaluate the efficiency of their solution. However, we can obviously notice that the spectrum of the considered attacks is much more restricted than the panorama of attacks that can be carried on a WBAN environment (see Section II.C). Thusly, trust management resiliency should get more interest to be able to propose more efficient solutions responding and resolving a large number of attacks. For that, an attack-free framework can be considered as an objective to build a viable trust management proposal.
6. Green trust and QoS. The trust management process induces a communication overhead and time complexity. These parameters should be considered for reliable performance evaluation. In fact, slight trust computation and fast data dissemination are crucial in the WBAN environment since it is characterized by its real time based applications. Thus, it is essential to swiftly derive accurate trust values. All this lead us to recommend building a lightweight trust management process that is adapted to the devices capabilities. To do so, the energy consumption of the proposed solution should be low. In this regard, ongoing researches have to pay more attention to evaluating the energy efficiency of the trust framework. This point is more relevant in the context of green-IoT deployment where we define a green communication across the network. The energy consumption is subtle since it is directly related to the WBAN availability property. Emerging technologies (evoked in point (3)) propose novel approaches to improve the energy efficiency methodologies that should be taken into account during the trust framework building.
7. Trust negotiation. To establish trust, all existing approaches propose to calculate trust values based on some formulas. To alleviate this process, further works can consider trust negotiation consisting in a process that lead participant to a common agreement. The required trust level is initially negotiated between different nodes. The process is based on exchanging a set of credentials to attest on the honesty of a node. When the exchanged credentials are more sensitive, the negotiated trust level is higher. Each node in the network will have a trust policy in which it defines the trust levels associated with the required credentials. The negotiation process will hold when a new device joins the network.
8. End to end approaches. The proposed approaches consider either the intra-WBAN architecture or the inter-WBAN architecture. It is worthy to consider approaches that integrate communications in these both architectures in the trust framework building, especially because of the related functioning of these two types of communications. In other words, intra-WBAN communications need the inter-WBAN communications to be able to deliver data to the destination. Hence, an end to end secure and trustworthy process is required to manage in a suitable way the trusty entities and to keep the data safe. This could be considered for the four categories already evoked (see point (2)): entity-based, data-based, location-based and hybrid approaches. A great attention should be drawn to consider the different requirements when we consider inter-WBAN communications. This architecture presents more requirements related to scalability since an inter-WBAN architecture includes different WBAN that may be distant, especially with an unknown pre-deployment topology. Moreover, the attack surface is larger in this case since more attacks can be carried on like tampering or node capture.

## 8. Conclusions

**Figure 8:**
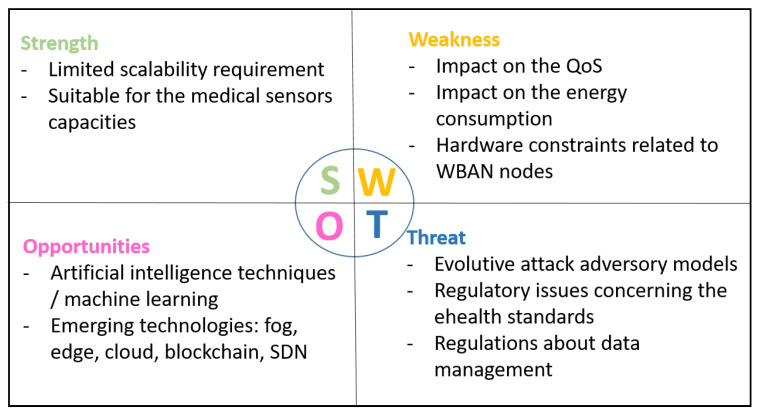
SWOT diagram for WBAN trust management.

The coronavirus epidemic has stressed the weaknesses of the healthcare systems all over the world. The use of WBAN in such context can be very relevant to save many patients’ lives and especially to remotely control the patient health and so to act at the opportune moment. This is only one example of the multiple use cases where using WBAN has a real added value. For that, we provide in this survey a review of the proposed approaches dealing with trust management in WBAN. First, we highlighted the trust management challenges for WBAN and classified the attacks on the trust management models. Then, we reviewed the existing intra- and inter-WBAN approaches. Moreover, we compared and classified them based on a new taxonomy to underline the weaknesses and the missing aspects that have to be taken into account for future researches. Furthermore, we gave a set of key points to be considered as the best practices to build a reliable and secure trust management framework. Finally, to conclude the paper, we propose in Figure 8 a SWOT diagram to outline the Strength, Weakness, Opportunities, and Threat of trust management in a WBAN environment. Table 7 gives the list of acronyms used along this paper.

## Figures and Tables

**Table 1:** Comparison of WSN and WBAN.

	Comparison Criteria	WSN	WBAN
Characteristics	Standard	IEEE 802.11.4	IEEE 802.15.6
	Topology	unchanged	changed
	number of nodes	hundreds	dozens
	scalability	large	small
	node size	no specific requirement	the smaller the better
	node energy	limited, replaceable	limited, irreplaceable
	Node lifetime	Months/years	the longer the better
	Mobility	low	high
	Implementation	Automatic; standalone	Human involvement
Requirements	Safety	depends on applications	very high
	Reliability	depends on applications	high
	Data rate	depends on applications	high
	Data loss	depends on applications	very high
	Data integrity	depends on applications	very high
	Biocompatibility	Not considered	considered

**Table 2:** Delay and bit rate requirements of healthcare data [6].

Data Source	Bit Rate (bps)	Delay (s)	Sampling Rate (Hz)
Electrocardiogram	10–100 k	<10	63–500
Blood pressure	10–30	>120	63
Non-invasive cuff	0.05	30–120	0.025
Cardiac output	1 k	<10	63
$CO_{2}$ concentration	1 k	30–120	63
Temperature (°C)	0.3	>120	0.02

**Table 3:** WBAN trust management requirements for different attacks.

	Requirements	Adaptive Behavior	Context Awareness	Reliability Reliability	Fair Treatment of New Users	Changing Identities	Trust Score of New Users
Attacks							
Ballot box stuffing		√		√			√
Bad mouthing attack (BMA)				√			
Collusion attack		√		√			
Whitewashing attack				√		√	
Sybil attack (SA)				√		√	
Newcomer attack				√	√	√	√
On/Off attack		√	√	√			
Traitor attack		√	√	√			
Grudge attack		√		√			

**Table 4:** Surveys related to the security in intra-WBAN.

Ref./Date	Trust Computation	Trust Update	Attack Patterns	Performance Evaluation	Validation Environment
[61]/2019	Weights correlated with the interaction trust, energy trust, data trust	Time driven	SA, BMA	Precision, recall, Throughput, PDR, E2E delay.	NS-2 and dataset from (http://guides.lib.berkeley.edu/publichealth/healthstatistics/rawdata)
[62]/2016	Collaborative filtering Most similar nodes. Resnick’s prediction [64]	Time driven	SA, BMA	Precision, Recall	GloMoSim 2.03
[65]/2019	Fixed values according to location: home or work or public location	Location based	DoS	Throughput, Packet Drop Percentage	Matlab
[66]/2017	The computations to build trust values is according to the opinion triangles represented by three variables and the consensus operator of the Subjective Logic	When packets are received	Routing attacks against RPL networks	Number of intruder detected. False Positive. False Negative. Undetected Positives Undetected Negatives	Matlab 100 × 100 square meters that involves 1000 nodes within 9 clusters
[67]/2015	Familiar network with high trust, Social network with middle trust and strangers’ network with low trust	When the network nodes are changing	Eavesdropping, Tracking, Replace, Spoofing attacks and Compromised sensor	Encryption time	Android-smart phone. ECC as asymmetric algorithm PHI size for AES 128 bits. PHI size for ECC 64 bits
[69]/2018	Based on the following node attributes: battery terminal voltage, receiver signal strength, and speed of movement	When nodes parameters change	N.A	Lifetime and throughput	Matlab 7.0. Nodes number: 100 nodes. Comparison to LEACH and QPRR algorithms
[71]/2019	The trust is calculated based on the data freshness and based on permissed delay for each data type	For each data forwarding	Malicious nodes	Delay	Matlab
[72]/2017	Based on a formula considering 3 parameters: member’s classification, Reliability trust, probability of health loss	Time and event driving	Malicious nodes	Mean Squared Error correct decision ratio Percentage of detection of malicious nodes	Environmental health IoT system. Members number: 100. Number of smart IoT devices: 100. Percentage of malicious nodesb ∈ [0, 30%]. Total simulation time = 20 h
[73]/2018	Based on indirect trust and applying a binomial distribution	The trust of i and j updated after each interaction between i and j	On-Off attack; Bad mouthing attack	Detection time Detection accuracy	Matlab
[76]/2012	The trust is calculated based on the direct, recommended and historical trust values. Each of them is calculated based on simple mathematical formulas and integrate the two parameters	Time driven	Malicious nodes	The average packet reception ratio (PRR)	Realworld experiment with multiple TelosB motes TelosB mote: 8 MHz CPU, 10 kb RAM, 48 kb ROM, and 802.15.4/ZigBee radio. The motes run TinyOS 2.1.0 Experiment duration: 500 s

**Table 5:** Surveys related to the security in inter-WBAN.

Ref./Date	Trust Computation	Trust Update	Attack Patterns	Performance Evaluation	Validation Environment
[77]/2017	The trust estimation is based on the direct and indirect trust	Node temperature changes	N.A.	packet drop ratio, packet delay, throughput and temperature under varying traffic conditions	N.A.
[78]/2016	The trust of a node is deduced from the opinion of all other nodes about it. All nodes of the network participate in defining the estimation of the node trust level	When initializing the routing protocol	Packet dropping attack	The throughput, the packet loss ratio and the end-to-end delay	NS-2. The mobile network with an average speed of 4 ms. The number of malicious nodes: 0–9
[79]/2017	The reputation value is based on the percentage of the number of transmitted and dropped packets	Time driving	Packet dropping attack	Throughput, Data privacy level, data aggregation efficiency	NS2. Routing protocol: Dynamic source routing protocol (DSR). Node density: 50, 100, 150, 200, 250, 300, 350, 400, 450, 500. Network area: 1500 × 1500 m
[82]/2018	The trust is calculated based on a fuzzy inference model and combining three parameters: the residual energy, the link expiration time and the received signal strength	When nodes parameters change	N.A	The nodes energy consumption, the latency, the delivery ratio, the network life span, and the model scalability	Matlab 2015b on ‘‘Intel Core i5 processor’’ with 2.30 GHz CPU and 8 GB RAM

**Table 6:** New classification of trust management approaches in WBAN

	Trust Objectives						Trust Properties						Trust Techniques						Trust Computation				
		Secure Routing	On-Off attack	malicious nodes	Dos attack	Packet dropping	Data privacy	Direct trust	Indirect trust	Historical trust	Objective trust	Subjective logic	Decentralized	Centralized	Bayesian Inference	Fuzzy logic	Simple formula	Collaborative filtering	Composition	Propagation	Aggregation	Update	Formation
Entity centric	[62]/2016			√					√									√			√	√	
	[66]/2017	√						√	√	√		√									√		
	[69]/2018												√				√		√				
	[73]/2018		√						√								√			√	√	√	
	[76]/2012				√			√	√	√				√			√				√	√	
	[77]/2017			√				√	√					√			√			√			
	[78]/2016			√							√						√				√		
	[82]/2018	√														√					√	√	
Data centric	[71]/2018			√													√					√	
Hybrid	[61]/2019			√				√					√							√	√	√	
	[67]/2015						√	√						√			√						
	[79]/2017						√	√					√				√				√		
Location centric	[65]/2019				√			√									√				√		
	[72]/2017			√										√					√				

**Table 7:** List of acronyms.

Acronym	Full-Form
AASR	Anonymous Secure Routing
ANODR	Anonymous On-Demand Routing
AODV	Ad Hoc On Demand Distance Vector
APs	Access Points
BAN-Trust	Body Area Network trust
BDTMS	Binomial Distribution-based Trust Management Scheme
BFTASR	biometric fusion based trusted anonymous secured routing protocol
BMA	Bad Mouthing Attack
BRSN	Beta Reputation System for Sensor Networks
CNTD	Clustered Neighbor Based Trust Dissemination
DODAG	Destination Oriented Directed Acyclic Graph
DoS	Denial of Service
DSR	Dynamic Source Routing protocol
E2E	End to end
FATD	Fault Aware Trust Determination
GDPR	General Data Protection Regulation
IEPSO	Improved Evolutionary Particle Swarm Optimization
IoT	Internet of Things
LEACH	Low Energy Adaptive Clustering Hierarchy
LPU	Local Processing Unit
MANETS	Mobile Adhoc Networks
MICS	Medical Implant Communication Service
NBTD	Neighbor Based Trust Dissemination
PDR	Packet Drop Ratio
PHI	Personal Health Information
PPM-HDA	Privacy-preserving and multifunctional health data aggregation
PRR	Packet Reception Ratio
QoS	Quality of Service
QPRR	QoS-Aware Peering Routing for Reliability
RDI-SSDA	Repute Derivative Incentive and Sparse Sampled Data Aggregation
RPM	Remote Patient Monitoring
RPL	Routing Protocol for Low-Power and Lossy Networks
SA	Sybil Attack
SAPA-BAN	Sybil Attack Prevention Algorithm for Body Area Networks
SDN	Software Defined Network
SGBAS	Self-adaptive Greedy Buffer Allocation and Scheduling algorithm
TBID	Trust-Based Intrusion Detection
TCBR	Trust Aware Cluster Based Routing Protocol
TRTMS	Time-window-based Resilient Trust Management Scheme
TTD	Tree Based Trust Dissemination
TTRP	Trust and thermal aware routing protocol
WBAN	Wireless Body Area Networks
WBSN	Wireless Body Sensor Network
WSN	Wireless Sensors Networks
